# Enhancer-Trap Flippase Lines for Clonal Analysis in the *Drosophila* Ovary

**DOI:** 10.1534/g3.114.010710

**Published:** 2014-07-14

**Authors:** Pamela Huang, Pankaj Sahai-Hernandez, Rudolf A. Bohm, William P. Welch, Bing Zhang, Todd Nystul

**Affiliations:** * Center for Reproductive Sciences, Departments of Anatomy and OB/GYN-RS, University of California, San Francisco, San Francisco, California 94143-0452; † Department of Biology, University of Oklahoma, Norman, Oklahoma 73019; ‡ Division of Biological Sciences, University of Missouri, Columbia, Missouri 65211; § Department of Biological and Health Sciences, Texas A&M University-Kingsville, Kingsville, Texas 78363-8202

**Keywords:** *Drosophila*, clonal analysis, Flp/FRT, ovary, stem cells

## Abstract

The *Drosophila melanogaster* genetic tool box includes many stocks for generating genetically mosaic tissue in which a clone of cells, related by lineage, contain a common genetic alteration. These tools have made it possible to study the postembryonic function of essential genes and to better understand how individual cells interact within intact tissues. We have screened through 201 enhancer-trap flippase lines to identify lines that produce useful clone patterns in the adult ovary. We found that approximately 70% of the lines produced clones that were present in the adult ovary and that many ovarian cell types were represented among the different clone patterns produced by these lines. We have also identified and further characterized five particularly useful enhancer-trap flippase lines. These lines make it possible to generate clones specifically in germ cells, escort cells, prefollicle cells, or terminal filament cells. In addition, we have found that *chickadee* is specifically upregulated in the posterior escort cells, follicle stem cells, and prefollicle cells that comprise the follicle stem cell niche region. Collectively, these studies provide several new tools for genetic mosaic analysis in the *Drosophila* ovary.

The *Drosophila* ovary is a tractable model for studying diverse biological processes, including oogenesis, tissue morphogenesis, and stem cell self-renewal. A common strategy for studying gene function in the ovary is to generate homozygous mutant cells by flippase (Flp)/FRT mitotic recombination. Often, Flp is expressed under the control of a heat shock promoter, which allows for precise control over the timing of clone induction, but it is not possible to target recombination to specific cell types or tissues with this method. Thus, mutant clones are generated in multiple cell types, making it more difficult to assess the cell-autonomous role of genes of interest. Another widely used approach has been to use a UAS-Flp construct and Gal4 lines that are expressed in specific tissues to activate Flp expression (Blair 2003; Duffy *et al.* 1998). This system has the disadvantage that two transgenes are required, and that Gal4 is sensitive to temperature and is subject to autoregulation and variegation. In addition, the available Gal4 lines that express in the germline are not very efficient at generating recombination in the germline. Recently, Bohm *et al.* (2010) generated a large collection of “enhancer-trap flippase” (ET-Flpx2) lines with random single insertions of a *P*-element containing two copies of Flp downstream from a minimal promoter (Figure 1B). For many of these lines, the expression of Flp is controlled by local enhancer elements, thus allowing for tissue and cell-type-specific mitotic recombination. The authors demonstrated that these lines can be used to obtain reproducible, specific clone patterns in the larval and adult brain, which also led to reproducible behavioral changes in flies. However, ovarian clone patterns have not been investigated.

**Figure 1:**
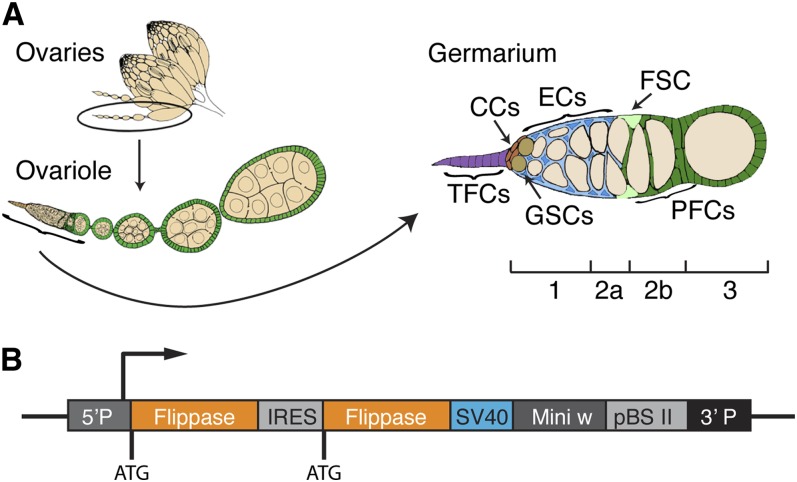
The *Drosophila* ovary and the ET-Flpx2 construct. (A) A diagram of the *Drosophila* ovary. Each ovary contains multiple ovarioles and each ovariole has a structure at the anterior tip called the germarium. Within the germarium are terminal filament cells (TFCs; purple) and cap cells (CCs; red), escort cells (ECs; blue), germline stem cells (GSCs; tan), germ cells (light tan), follicle stem cells (FSCs, light green), prefollicle cells (green), and follicle cells (green). The germarium is divided into four regions: 1, 2a, 2b, and 3, as indicated. (B) The ET-Flpx2 construct contains two Flp genes in tandem separated by an IRES site, an SV40 termination sequence, and mini-white.

The adult ovary contains multiple cell types (Figure 1A). Each ovary is composed of individual strands of developing follicles called ovarioles, and each ovariole contains a structure at the tip called the germarium that is the source of new follicle production. Germaria are divided into four morphologically distinct regions, region 1, 2a, 2b, and 3, that correspond to stages of early germ cell development. New germ cells are produced during adulthood by two to three germline stem cells (GSCs) that reside within a niche at the anterior tip of the germarium (region 1). GSC niche signals are produced by nearby terminal filament cells and cap cells, and both GSCs and their daughters are surrounded by a population of thin stromal cells called escort cells (Losick *et al.* 2011; Xie 2013). GSC daughters undergo four mitotic divisions to become a 16-cell cyst as they move away from the niche and past the escort cells in regions 1 and 2a. Each germarium also contains exactly two follicle stem cells (FSCs) that reside in niches at the region 2a/2b border and produce prefollicle cells (Sahai-Hernandez *et al.* 2012). As the germ cell cysts move from region 2a to 2b, they become encapsulated by prefollicle cells, which associate with the germ cells and begin to differentiate gradually, over the course of several cell divisions (Franz and Riechmann 2010; Chang *et al.* 2013). As prefollicle cells move through regions 2b and 3, they differentiate into main body follicle cells, which comprise the majority of the follicular epithelium; stalk cells, which connect adjacent follicles to each other; or polar cells, which reside along the anterior/posterior axis of the follicles and provide positional cues.

The GSCs and FSCs are the only cell types that both remain in the germarium long-term and regularly proliferate during adulthood. Thus, they can be easily targeted using an FRT clone generation system, such as MARCM (Lee and Luo 2001) (for somatic cells) or a negatively-marked clone system (Xu and Rubin 1993) in which one chromosome must be asymmetrically segregated during mitosis, and a heat-shock Flp to induce recombination specifically during adulthood. In contrast, these methods are not well-suited for targeting specific cell populations (such as germ cells but not somatic cells) or cell populations that divide rarely, such as escort cells and terminal filament cells. Therefore, we screened through a collection of 201 ET-Flpx2 lines (Bohm *et al.* 2010) for lines that produce ovarian clones with patterns that are difficult to obtain using existing methods.

Here, we report the results of the screen and highlight five lines that generate clones within specific subpopulations of cells in the ovary. We have verified the clone patterns for each of these five lines and mapped the ET-flpx2 insertion site for four lines. Furthermore, one line led us to the discovery that *chickadee* (*chic*) is specifically upregulated in the FSC niche region. Finally, we provide a summary of the information for all 201 lines, which can be used as a resource for selecting additional lines of interest.

## Materials and Methods

### Fly stocks and crosses

All stocks and crosses were maintained on standard molasses food at room temperature or 25°. In addition to the 201 ET-flpx2 lines, the following stocks were used:

1. yw, actinP > cd2 > gal4; UAS-gfp
2. yw, P{Act5C(FRT.polyA)lacZ.nls1}^3^, ry506
3. w; Act-FRT-stop-FRT-lacZnls, Ubi-FRT-stop-FRT-GFPnls; Act-FRT-stop-FRT-GAL4, UAS-his2A::RFP/SM5-TM6B (TIE-DYE)
4. hsFlp; FRT 40a, Ubi-GFP
5. w; FRT40a

To obtain flies for ovary dissections, crosses were set up between the ET-flpx2 lines and one of the reporter stocks listed above. Once the progeny began to eclose, all adults were discarded and newly eclosed progeny were maintained on molasses food with fresh wet yeast daily for 2 d or 7 d.

### Immunofluorescence

Ovaries were dissected in 1× Graces Medium with forceps, fixed in 4% paraformaldehyde, rinsed in 1× PBS plus 0.2% Tween (PBST), incubated overnight at 4° with primary antibody, washed for 1 hr with PBST, incubated at room temperature with secondary for 2 hr, washed for 1 hr with PBST, and incubated with PBS plus 1 ng/µl DAPI. Ovaries were then mounted on glass slides in vectashield and viewed using a Zeiss Axioimager for scoring and imaged with a Zeiss Apotome to obtain optical sections. The following primary antibodies were used: mouse anti-GFP (1:1000; Invitrogen), rabbit anti-GFP (1:5000; Torrey Pines Biolabs), rabbit anti-vasa (1:1000; Santa Cruz Biotechnology), mouse anti-LacZ (1:1000; Promega), rabbit anti-LacZ (1:100; Cappel), and mouse anti-Fas3 (1:50) and mouse anti-chickadee (1:100) from the Developmental Studies Hybridoma Bank. The following secondary antibodies were used: anti-rabbit and anti-mouse conjugated to Alexafluor 488, 546, or 555 (1:1000; Invitrogen).

### Clone generation

To generate FSC clones in flies with the *hsFlp* construct, flies of the appropriate genotype were cultured at 25° for at least 2 d after they eclosed, heat shocked at 37° four times over 2 d for 1 hr per heat shock, and cultured for at least 5 d to allow transient follicle cell clones to exit the germarium. Flies were given fresh wet yeast daily throughout the culture periods.

### Splinkerette

Splinkerette PCR was performed according to previously established protocol (Potter and Luo 2010). Generally, DNA was extracted from 10 flies per line, digested with BstY1 restriction enzyme, ligated to a double-stranded splinkerette oligonucleotide with a stable hairpin loop and compatible sticky ends, and run through two PCR cycles with nested primers (one primer for the splinkerette and one primer specific for the *P*-element). Oligonucleotide sequences were as follows:SPLNK-GATC-TOP GATCCCACTAGTGTCGACACCAGTCTCTAATTTTTTTTTTCAAAAAAASPLNK-BOT CGAAGAGTAACCGTTGCTAGGAGAGACCGTGGCTGAATGAGACTGGTGTCGACACTAGTGG SPLNK#1 CGAAGAGTAACCGTTGCTAGGAGAGACCSPLNK#2 GTGGCTGAATGAGACTGGTGTCGAC3′SPLNK#1 CACTCAGACTCAATACGACAC3′SPLNK#2 GGATGTCTCTTGCCGAC3′SPLNK-SEQ CGGGACCACCTTATG

PCR products were separated on a 1% agarose gel stained with ethidium bromide. Single bands were excised from the gel, and the DNA was purified and sequenced. Initial splinkerette PCR reactions for lines 168 and 688A both indicated a *P*-element insertion at X:19586247, which is likely to be the original insertion site of the ET-Flpx2 before mobilization. Moreover, genetic analysis indicated that these lines both contain ET-Flpx2 insertions on an autosome. Therefore, we crossed away the X chromosome and redid the splinkerette reactions on the resulting *w^mW+^* flies. However, after multiple attempts using splinkerette PCR, we were still unable to obtain reliable information about the autosomal insertion sites in these two lines.

### Inverse PCR

Genomic DNA from lines 168 and 688A was sent to Best Gene, Inc. (www.thebestgene.com), where inverse PCR was attempted according to standard procedures (http://www.fruitfly.org/about/methods/inverse.pcr.html). Multiple attempts to map the insertion in line 688A were unsuccessful, but two independent iPCR experiments mapped line 168 to an insertion site in the *krasavietz* gene. The genomic sequence returned by one of these iPCR experiments is provided below.

TGTTGCCACCTTTTGGACACGTTGCTGAGCTCTGTACCATTGCGTGCGATCTGTGCCACGGTCTTACAGCCAGGCATCGGTAATAAGCAGCCCAACAGCTACGCAGCAAGTTACTTCTATTCGCAGCAAAACAGATTTTTTTGTTTTAATCGTAAGTATAGGAGTGAAAAATAGCGCTAGAGTAGACCTATGTACCCAGAAAGAACAATAGGGCGAGTAAAAACGCGGTCGGGGACATTTCTCTGGGCTTGGGCAATCCTTTGGGTGCGCTTTCGTTGGAAAAGGGGTTATGTACGAACTGAGGGGGTACGTAAGGCCTGATTACGCACCGGGCGGCCATTTATTCATGCAAAAAATCATTTGGTGGGCGTAGGCCTTTGTTCGGGTGGACCTTGTTCGTTATTTAGTAAGCGGGACAGCAATATACACACTTTGAACCCCCATCCCACATTTTTTCTCACCGCCTCCCTCTAATCTCCGTGTTCCCTGTGACCATCATTCCGCTCTC

## Results

**Figure 2:**
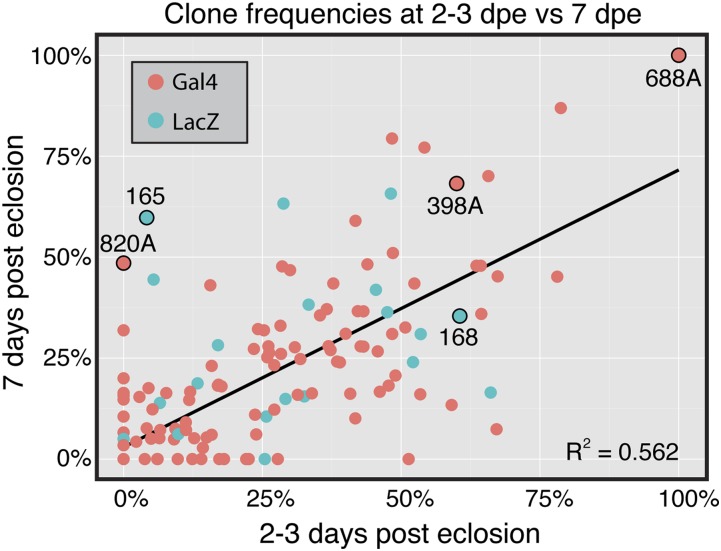
Clone frequencies in the germarium at 2 to 3 d *vs.* 7 d after eclosion. A scatter plot showing the relationship between the overall clone frequency at 2 to 3 d and 7 d after eclosion in the germarium for all 201 lines analyzed. If fewer than five germaria had a clone at a given time point, the total number of germaria observed was not recorded and the frequency was rounded down to 0.

To identify ET-Flpx2 lines that produce clones in the ovary, we crossed 201 lines to either an *Act5C > stop > LacZ* (Struhl and Basler 1993) or *actinP > cd2 > gal4*; *UAS-GFP* (Pignoni and Zipursky 1997) reporter stock. We then examined clone patterns by immunofluorescence in the germaria from the F1 progeny, first at 2 to 3 days after eclosion (dpe) to identify clone patterns that likely arose during development and then again at 7 dpe to identify clone patterns that persisted or were newly generated during adulthood (Supporting Information, Table S1). We observed a similar clone frequency with both reporters (Table 1) and found that, overall, 69.1% (139/201) of the lines produced clones in germarium (Table 2). Moreover, we observed a correlation between the frequency of germaria with clones in the F1 progeny from each line at 2 to 3 d and 7 d after eclosion (Figure 2), suggesting that clones were often produced during development and persisted into adulthood. Most lines that produced ovarian clones generated clones in multiple ovarian cell types, and many of these produced clones only sporadically or in a low percentage of ovarioles. However, we identified five lines that produced particularly useful or informative clone patterns. For each of these lines, we repeated the crosses and quantified the clone patterns and frequencies throughout the ovariole (rather than just in the germarium, as we did in the initial screen) at 2 and 7 dpe. The results of this analysis are described below.

**Table 1:** Frequency of cell types labeled by the ET-Flpx2 lines in this study

Type	Any Cell Type	Cap or Terminal Filament	Escort	Follicle (2–3 dpe)	FSC (7 dpe)	Germ Cells
All	69.1% (139/201)	51.8% (72/139)	95.7% (133/139)	67.6% (94/139)	57.6% (80/139)	N/A
LacZ	66.7% (22/33)	81.8% (18/22)	86.4% (19/22)	77.3% (17/22)	59.0% (13/22)	50.0% (11/22)
Gal4	70.1% (117/167)	46.2% (54/117)	97.4% (114/117)	65.8% (77/117)	57.3% (67/117)	N/A

**Table 2:** Summary of clone frequencies for all 201 ET-Flpx2 lines

Day	Frequency of Lines with Clones in the Germarium	Average Frequency of Germaria-Labeled/Slide (from Lines with Clones)	Number of Germaria Scored (from Lines with Clones)
2 and 7	69.1%	28.8%	20,093
2	60.0%	31.0%	10,089
7	64.5%	26.7%	10,004

### Germ cell clones

**Figure 3:**
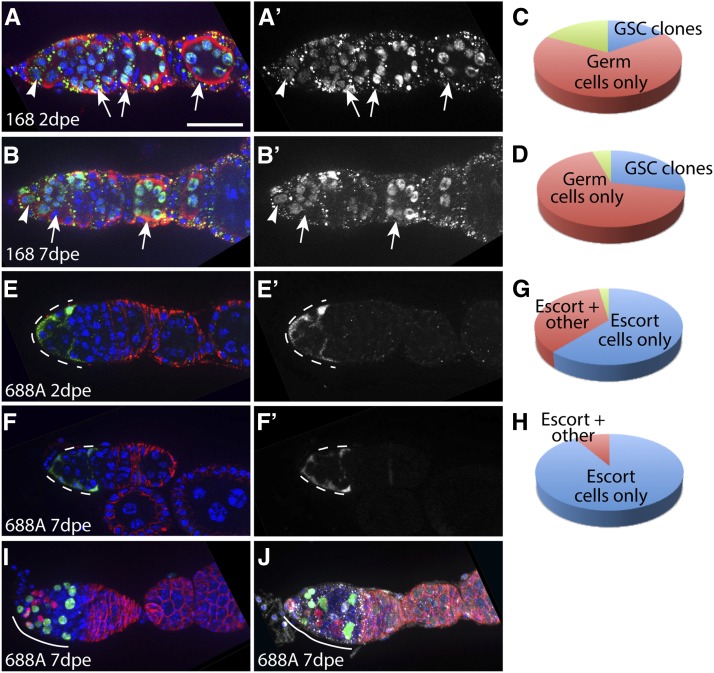
Clone patterns and frequencies produced by lines 168 and 688A. Line 168 crossed to the LacZ Flp reporter frequently produces clones in the germline but not somatic cells, and line 688A crossed to the Gal4 Flp reporter frequently produces clones in escort cells. Germaria from line 168 at 2 d (A) or 7 d (B) after eclosion (dpe) with GSC clones stained for vasa (red), LacZ (clone label, green), and DAPI (blue). Arrowheads indicate labeled GSCs and arrows indicate labeled germ cell cysts. Pie charts show the proportion of ovarioles with one or more GSCs and multiple germ cells but no somatic cells labeled (blue), multiple germ cells but no GSCs or somatic cells (red), or GSCs and/or germ cells as well as somatic cells (green) at 2 dpe (C) and 7 dpe (D). Germaria from line 688A with clones in escort cells (dashed white lines) at 2 dpe (E) or 7 dpe (F) stained for FasIII (red), GFP (clone label, green), and DAPI (blue). Pie charts show the proportion of ovarioles with clones only in escort cells (blue), escort cells and other cell types (red), or no clones (green) at 2 dpe (G) and 7 dpe (H). (I and J) Germaria with TIE-DYE escort cell clones produced by line 688A at 7 dpe and stained for FasIII (red), LacZ (white), and DAPI (blue). The RFP (red) and GFP (green) clone markers were detected using the native fluorescence signal from these proteins. Small dots visible in (A) and (B) and (J) are background staining from the LacZ antibody. Anterior is to the left. Scale bar indicates 10 µm.

We found that line 168 crossed to the LacZ Flp reporter frequently produced clones specifically in the germline (Figure 3, A–D). At 2 dpe, all ovarioles (100%, n = 57) contained at least one germ cell clone, and 17.5% (n = 10/57) of these ovarioles contained GSC clones. In total, 82.4% (38 out of 47 ovarioles with germ cell clones and 9 out of 10 ovarioles with GSC clones) had no somatic cell clones. Most of the remaining ovarioles (n = 9/10) had both germ cell or GSC clones and terminal filament cell clones, and one ovariole had clones in the germ cell, terminal filament cell, escort cell, and FSC populations. At 7 d after eclosion, again, all ovarioles (100%, n = 42) contained at least one germ cell clone, and 28.6% (n = 12/42) of these ovarioles contained GSC clones. Only two of these 42 ovarioles had somatic cells labeled as well; one contained a single labeled escort cell and one contained a single labeled cap cell. Given the efficiency with which this line produces clones in the germline (while also minimizing clones in the ovarian somatic cells), this line may be particularly useful in combination with the OvoD system that is used to generate embryos lacking the maternal contribution of a gene of interest (Perrimon 1984). The ET-Flpx2 insertion in line 168 maps to a site within *krasavietz* on chromosome 3, and we found that it is homozygous lethal (Table 3).

**Table 3:** Insertion sites and phenotypes of selected ET-Flpx2 lines

Line Number	Closest Gene	Insertion Site	Chromosome	Orientation	Phenotype of Homozygous Insertion
165	*Ect4*	3L:8065115	3	—	Viable
168	*krasavietz*	3R:1426379	3	+	Lethal
398A	*chickadee*	2L:5977570	2	+	Viable
688A	ND	ND	3	N.D.	Lethal
820A	*cAMP-dependent protein kinase R2*	2R:5911366	2	+	Viable

### Escort cell clones

We found that line 688A crossed to the Gal4 Flp reporter produced clones of every type that we screened for and that it produced a particularly high frequency of escort cell clones (Figure 3, E–J). Moreover, the escort cell clones produced by line 688A tended to be bigger than the escort cell clones produced by many other lines. In addition, line 688A frequently produced ovarioles in which escort cells were the only somatic cell type labeled. Specifically, we found that, at 2 dpe, 97.5% (n = 79/81) of ovarioles had four or more escort cells labeled, and 60.8% of these ovarioles (n = 48/79) did not have any other somatic cell type labeled. By 7 dpe, 100% of the ovarioles had four or more escort cells labeled and 91% (n = 51/56) had no other somatic cell type labeled, whereas the remaining 9% (n = 5/56) had both escort cell and FSC clones.

To further investigate the escort cell clones produced by line 688A, we used the multi-color TIE-DYE clone system (Worley *et al.* 2013). This clone system utilizes three separate clonal marking constructs to label cells, an *Act FRT-stop-FRT lacZ^NLS^* (Struhl and Basler 1993), a *Ubi FRT-stop-FRT eGFP^NLS^* (Evans *et al.* 2009), and an *Act FRT-stop-FRT GAL4* (Pignoni and Zipursky 1997) combined with a UAS-His2A::mRFP (Emery *et al.* 2005). On expression of Flp, recombination can occur in one or more of these clonal marking constructs, which allows for a wide variety of clonal labeling patterns. This is useful because it makes it possible to identify independent clones with different clonal labeling patterns, even when they are adjacent to one another. We found that most germaria contained two to three large independent escort cell clones at 7 dpe, indicating that Flp expression begins during the expansion of the escort cell lineage. Specifically, because escort cells rarely divide during adulthood (Morris and Spradling 2011; Kirilly *et al.* 2011), these clones were likely produced during pupal development. In addition, we noticed LacZ^+^ germ cell clones with the TIE-DYE clone system, indicating that line 688A also expresses Flp in the germline. We mapped the *P*-element insertion in this line to the third chromosome and found that it is homozygous lethal (Table 3).

### Prefollicle cell clones

**Figure 4:**
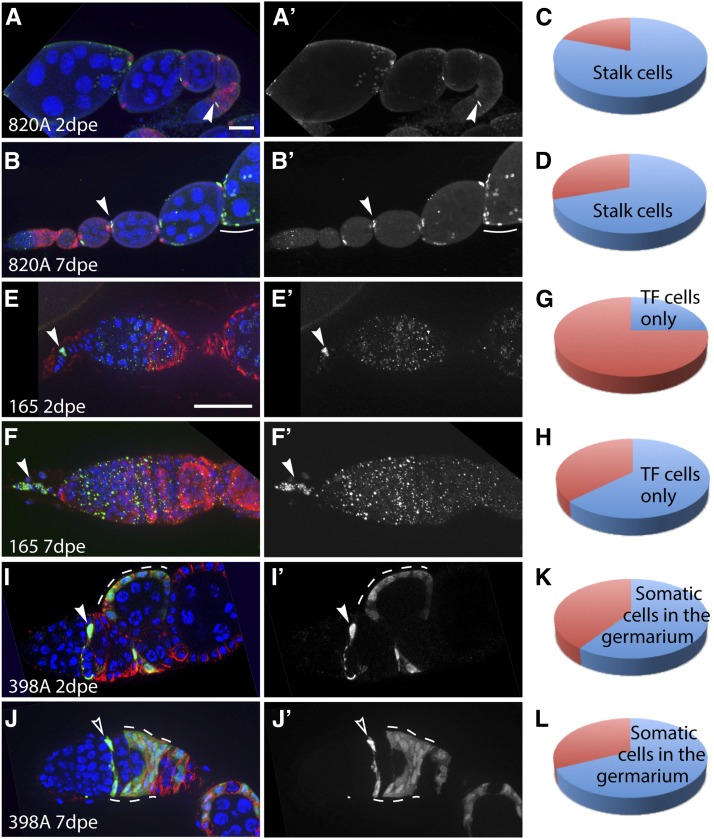
Clone patterns and frequencies produced by lines 820A, 165, and 398A. Line 820A crossed to Gal4 Flp reporter frequently produces clones in stalk cells, polar cells, and prefollicle cells. Line 165 crossed to LacZ Flp reporter produces clones specifically in terminal filament cells. Line 398 crossed to Gal4 Flp reporter frequently produces clones in escort cells, FSCs, and prefollicle cells. Germaria from line 820 at 2 d (A) or 7 d (B) after eclosion (dpe) with stalk cell clones stained for FasIII (red), GFP (clone label, green), and DAPI (blue). Arrowheads indicate labeled stalk cells, and arrow indicates labeled polar/stalk precursors in the germarium. Pie charts show the proportion of ovarioles with labeled stalk cells (blue) or no clones (red) at 2 dpe (C) and 7 dpe (D). Germaria from line 165 at 2 dpe (E) or 7 dpe (F) with clones specifically in terminal filament cells stained for FasIII (E; red) or vasa (F; red), LacZ (clone label; green), and DAPI (blue). Arrowheads indicate labeled terminal filament cells. Pie charts show the proportion of ovarioles with labeled terminal filament cells (blue) or no clones (red) at 2 dpe (G) and 7 dpe (H) dpe. Germaria from line 398 at 2 dpe (I) or 7 dpe (J) stained for FasIII (red), GFP (clone label, green), and DAPI (blue). In (I), a labeled prefollicle cell is indicated with a solid arrowhead, and a large follicle cell clone that likely originated in the prefollicle cell region is indicated with a dashed white line. In (J), a labeled escort cell is indicated with an open arrowhead and an FSC clone is indicated with dashed white lines. Pie charts show the proportion of ovarioles with labeled somatic cells in the germarium (blue) or no clones (red) at 2 dpe (K) and 7 dpe (L). Small dots visible in (E) and (F) are background staining from the LacZ antibody. Anterior is to the left. Scale bar indicates 10 µm.

We found that line 820A crossed to the Gal4 Flp reporter frequently induced clones in prefollicle cells within region 2b during adulthood, including the precursors of main body follicle cells, polar cells, and stalk cells (Figure 4, A–D). At 2 dpe, 80.3% (n = 61/76) of the ovarioles had labeled stalk cells, but very few clones were present in the germaria. In the initial screen, we found fewer than five germaria with clones at 2 to 3 dpe. In the rescreening of this line, we noticed that clones outside of the germarium typically included one or more cells within the stalk plus a small cluster of polar and/or follicle cells on the anterior of the follicle at the point of stalk attachment. At 7 dpe, 64.9% (n = 113/174) of ovarioles had labeled stalk cells and, again, clones typically included anterior follicle cells and polar cells. However, at 7 dpe, we also frequently observed labeled prefollicle cells in region 2b of the germarium (Figure 4A, arrowhead) and occasionally observed FSC clones. Collectively, these patterns suggest that Flp is expressed in early prefollicle cells that are the progenitors of the stalk cells as well as the polar cells and main body follicle cells that colonize the anterior portion of the follicle. We mapped the ET-Flpx2 insertion in line 820A to a site within *cAMP-dependent protein kinase R2* on chromosome 2 (Table 3).

### Terminal filament clones

We found that line 165 crossed to the LacZ Flp reporter produced clones exclusively in terminal filament cells (Figure 4, E–H). At 2 dpe, 25.0% (n = 14/56) of the ovarioles had one or more labeled terminal filament cells, and the remaining 75.0% of ovarioles had no labeled cells at all. By 7 dpe, 63.3% (n = 31/49) of the ovarioles had one or more terminal filament cells labeled and, again, no other clones were observed. We mapped the ET-Flpx2 insertion in line 165 to a site within *ECT4* on chromosome 3 (Table 3).

### The FSC niche region

**Figure 5:**
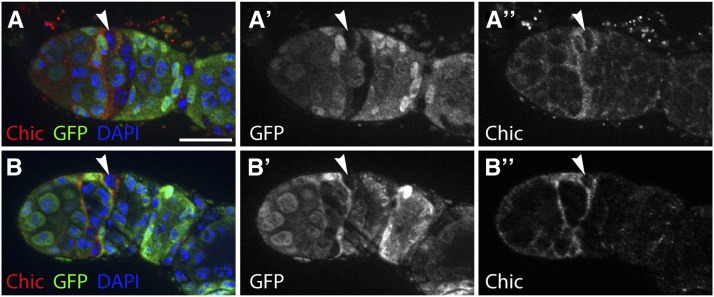
Chickadee is a marker of the FSC niche region. (A and B) Germaria with a mature GFP^−^ FSC clone stained for chickadee (red), GFP (clone marker; green), and DAPI (blue). Arrowhead indicates the FSC, which can be identified as the anterior-most cell in the clone. Anterior is to the left. Scale bar indicates 10 µm.

Line 398A crossed to the Gal4 Flp reporter produced somatic cell clones in the germarium in 60.0% (n = 57/95) and 68.2% (n = 58/85) of ovarioles at 2 dpe and 7 dpe, respectively. Specifically, we noticed that many of these clones were within the FSC niche region, which includes escort cells in region 2a, FSCs, and prefollicle cells in region 2b (Figure 4, I–L). We found that the ET-Flpx2 was inserted at a site within *chic* (Table 3), which has well-characterized roles in *Drosophila* oogenesis (Verheyen and Cooley 1994; Cooley *et al.* 1992; Jackson and Berg 2002; Baum and Perrimon 2001). We stained for chic in wild-type ovaries and found, consistent with previous studies, that chic is expressed in somatic cells throughout the germarium. In addition, we noticed that chic is specifically upregulated in a narrow strip of somatic cells at the region 2a/2b border. This expression pattern was very consistent, occurring in 100% (n = 100) of the germaria we examined. To determine whether the subset of cells with high levels of chic expression included the FSCs, we generated FSC clones in adult flies using *hsFlp* and a GFP^−^ clonal marking system and stained for chic (Figure 5). Because escort cells divide infrequently during adulthood, they are rarely labeled by this method. Therefore, by 5 d after clone induction, FSCs can be reliably identified as the anterior-most cell in a GFP^−^ clone. In all of the germaria we examined with mature FSC clones (n = 21), *chic* was upregulated in posterior escort cells, FSCs, and early prefollicle cells near the region 2a/2b border (Figure 5). This indicates that chic is a highly sensitive and specific marker for the FSC niche region within the germarium.

## Discussion

Our screen has identified a new set of lines for the study of oogenesis and stem cell biology in the *Drosophila* ovary. Previous screens identified LacZ and Gal4 enhancer trap lines with expression in the ovary (Grossniklaus *et al.* 1989; Manseau *et al.* 1997; Fasano and Kerridge 1988), and several of these lines have become standard, versatile tools for a wide range of studies. LacZ enhancer traps have helped to identify distinct ovarian cell types, whereas Gal4 enhancer trap lines have made it possible to both label and manipulate gene expression in distinct subsets of cells.

Clones induced by FRT recombination can also be used to identify cell types, such as stem cells, as well as to manipulate gene expression. Because the ET-Flp2x lines combine the features of an enhancer trap with Flp expression, they can be used much like Gal4/UAS-Flp combinations have been used previously. However, compared to existing tools, the ET-Flp2x lines have a distinct set of drawbacks and advantages. For example, the timing of Flp expression in ET-Flp2x lines cannot be controlled as it can be by temperature shifting flies with Gal80^ts^/Gal4/UAS-Flp or hsFlp. However, the ET-Flp2x lines still allow for spatially restricted clone induction while freeing the Gal4/UAS system for other uses. In addition, if the goal is to induce transgene expression in a subset of cells, then generating clones with an ET-Flp2x line may be a useful alternative to using a Gal4 enhancer trap in some cases because expression can be driven throughout the clone by a strong, uniform driver such as tub-Gal4 or ubi-Gal4 even if the ET-Flp2x line that provides the desired spatial specificity has relatively weak or variable expression levels.

Another advantage of ET-Flpx2 lines is the ability to label a group of cells in the adult tissue that shares a common progenitor. This is useful for studying the developmental relationships between cell types and for labeling subsets of cells within a uniform population. For example, line 688A may be useful for investigating whether there is functional diversity in the escort cell population. As a population, escort cells perform diverse functions in germ cell development and FSC self-renewal (Liu *et al.* 2010; König *et al.* 2011; Kirilly *et al.* 2011; Eliazer *et al.* 2014; Sahai-Hernandez and Nystul 2013) but, in many cases, it is unclear which escort cells perform these functions. This has been difficult to address, in part, because there are no Gal4 lines available with partial expression in the escort cell population. However, with line 688A, it is now possible to label and genetically manipulate subsets of developmentally related escort cells. Moreover, because line 688A labels different subsets of escort cells in different germaria, the same flies could be used to compare, for example, the phenotypes caused by knocking down a gene of interest in region 1 escort cells *vs.* region 2a escort cells. Likewise, line 820A will be useful for addressing questions about the specification of the polar/stalk lineage (Nystul and Spradling 2010).

Finally, as with other types of enhancer traps, the ET-Flpx2 lines can be used to identify genes with interesting expression patterns. For example, our studies of line 398A led to the discovery that *chic* is specifically upregulated in the FSC niche region. This establishes chic as a useful marker and suggests that it may be important for the function of the FSC niche. Chic is the *Drosophila* homolog of profilin (Cooley *et al.* 1992), which facilitates actin polymerization by promoting the recycling of ADP-bound actin monomers and aiding in the recruitment of actin monomers to the ends of actin filaments (Dominguez and Holmes 2011). Escort cells and prefollicle cells in the FSC niche region are highly dynamic and likely undergo continual cytoskeletal remodeling to facilitate the passage of each germ cell cyst and promote FSC self-renewal (Morris and Spradling 2011; Kirilly *et al.* 2011; Sahai-Hernandez and Nystul 2013). It will be interesting to investigate whether chic functions in these cells to promote these dynamic aspects of the FSC niche.

The lines we have described here complement existing tools for genetic mosaic analysis in *Drosophila* (Hafezi and Nystul 2012). Although we have thus far only tested these lines with “flip-out” clone generation systems, they could be combined with any of the standard Flp/FRT-based tools for driving transgene expression or generating homozygous mutant cells. In addition, as we showed for line 688A, they can be combined with newer multi-color clone generation systems to obtain additional information about lineage relationships. Moreover, our screen is part of a larger collaborative effort to characterize the ET-Flpx2 collection and our results will be compared with the results from two currently ongoing screens for ET-flpx2 lines that produce clones in the larval and adult brains and wing discs. Collectively, the ET-Flpx2 lines will be useful for a wide range of studies aimed at understanding cellular function within a multi-cellular, *in vivo* context.

## Supplementary Material

Supporting Information
