# Supplementary material for: Enhancer-Trap Flippase Lines for Clonal Analysis in the Drosophila Ovary
Source: G3 (Bethesda). 2014 Jul 14;4(9):1693–9. doi: 10.1534/g3.114.010710 (PMC4169162; doi:10.1534/g3.114.010710)
Supplement: Supporting Information [file supp_4_9_1693__index.html]

Enhancer-Trap Flippase Lines for Clonal Analysis in the Drosophila Ovary — Supporting Information 

# Enhancer-Trap Flippase Lines for Clonal Analysis in the *Drosophila* Ovary

## Supporting Information for Huang *et al.*, 2014

**Files in this Data Supplement:**

- Figure S1 - Identification of insertion sites for selected lines. (PDF, 337 KB)
- Table S1 - Summary of clone patterns for 201 ET-Flpx2 lines. (.xlsx, 60 KB)
