# Supplementary material for: Enhancer-Trap Flippase Lines for Clonal Analysis in the Drosophila Ovary
Source: G3 (Bethesda). 2014 Jul 14;4(9):1693–9. doi: 10.1534/g3.114.010710 (PMC4169162; doi:10.1534/g3.114.010710)
Supplement: Supporting Information [file supp_g3.114.010710_010710SI.pdf]

G3·Genes | Genomes | Genetics

Enhancer-trap Flippase Lines for Clonal Analysis in the *Drosophila* Ovary

Pamela Huang[1*], Pankaj Sahai-Hernandez[1*], Rudolf A. Bohm[4], William P. Welch[2], Bing Zhang[2,3], and Todd Nystul[1§]
*These authors contributed equally to this work.
[§]Corresponding author

[1]*Center for Reproductive Sciences*
Departments of Anatomy and OB/GYN-RS
University of California, San Francisco
San Francisco, CA 94143-0452
todd.nystul@ucsf.edu

[2]*Department of Biology*
University of Oklahoma
Norman, OK 73019

[3]*Division of Biological Sciences*
University of Missouri
Columbia, MO 65211

[4]*Department of Biological and Health Sciences*
Texas A&M University-Kingsville
Kingsville, Texas 78363-8202

DOI: 10.1534/g3.114.010710

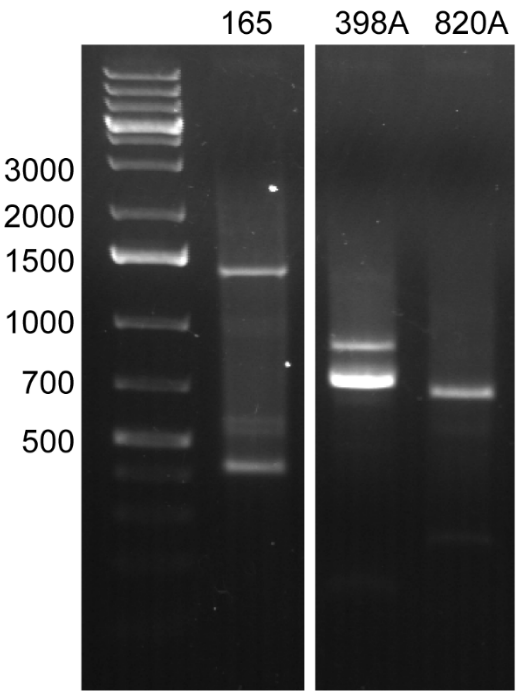

Figure S1 Identification of insertion sites for selected lines. Splinkerette PCR was performed to amplify the genomic regions flanking the ET-Flpx2 insertion sites in lines 165, 398A, and 820A. Bands were excised, gel purified and sequenced, and sequence was aligned to the *Drosophila* genome to determine the insertion site.

Table S1 Summary of clone patterns for 201 ET-Flpx2 lines

Available for download as an Excel file at http://www.g3journal.org/lookup/suppl/doi:10.1534/g3.114.010710/-/DC1
